# Requirements and special considerations for drug trials with children across six jurisdictions: 2. Ethics review in the regulatory approval process

**DOI:** 10.3389/fmed.2025.1539787

**Published:** 2025-02-11

**Authors:** Breanne Stewart, Pirkko Lepola, Gunter F. Egger, Fahimeda Ali, Albert J. Allen, Alysha K. Croker, Andrew J. Davidson, Pamela Dicks, Saul N. Faust, Dionna Green, Collin Hovinga, Agnes V. Klein, Robyn Langham, Hidefumi Nakamura, Laura Pioppo, Shiva Ramroop, Michiyo Sakiyama, Isabel Sanchez Vigil de la Villa, Junko Sato, Donna L. Snyder, Mark A. Turner, Sarah Zaidi, Kanecia Zimmerman, Thierry Lacaze-Masmonteil

**Affiliations:** ^1^Maternal Infant Child and Youth Research Network (MICYRN), Vancouver, BC, Canada; ^2^Department of Children and Adolescents, University of Helsinki and Helsinki University Hospital, Helsinki, Finland; ^3^Pediatric Medicine Office and Data Analytics and Methods Task Force, European Medicines Agency (EMA), Amsterdam, Netherlands; ^4^Paediatrics – Innovative Medicines, Healthcare Quality & Access, MHRA, London, United Kingdom; ^5^Institute for Advances Clinical Trials for Children, Rockville, MD, United States; ^6^Critical Path Institute, Tucson, AZ, United States; ^7^Biologic and Radiopharmaceutical Drugs Directorate, Health Products and Food Branch, Health Canada, Ottawa, ON, Canada; ^8^Melbourne Children’s Trials Centre, Murdoch Children’s Research Institute, Melbourne, VIC, Australia; ^9^NHS Scottish Children’s Research Network, Royal Aberdeen Children’s Hospital, Aberdeen, United Kingdom; ^10^NIHR Southampton Clinical Research Facility and Biomedical Research Centre, University Hospital Southampton NHS Foundation Trust, Southampton, United Kingdom; ^11^Faculty of Medicine and Institute for Life Sciences, University of Southampton, Southampton, United Kingdom; ^12^Office of Pediatric Therapeutics, Office of the Commissioner, United States Food and Drug Administration, Silver Spring, MD, United States; ^13^Therapeutic Goods Administration, Australian Government Department of Health and Aged Care, Canberra, ACT, Australia; ^14^National Centre for Child Health and Development, Department of Research and Development Supervision, Tokyo, Japan; ^15^Pediatric Drug Working Group, Pharmaceuticals and Medical Devices Agency, Tokyo, Japan; ^16^WCG Clinical, Princeton, NJ, United States; ^17^Department of Women’s and Children’s Health, University of Liverpool, Liverpool, United Kingdom; ^18^conect4children Stichting, Utrecht, Netherlands; ^19^Duke Clinical Research Institute, Duke University School of Medicine, Durham, NC, United States; ^20^Department of Pediatrics, Duke University School of Medicine, Durham, NC, United States

**Keywords:** pediatrics, clinical trials, regulatory science, research ethics review, research ethics committee, institutional review board

## Abstract

**Background:**

Conducting clinical drug trials (CTs) with children presents several challenges. A major challenge is the need to enroll participants at multiple sites across different jurisdictions. Recruiting the required number of children within a reasonable timeframe requires the study to be reviewed by Research Ethics Boards (REB) or Institutional Review Boards (IRB) at multiple sites across various jurisdictions. This work, undertaken by the Working Group (WG) on International Collaborations at the European Network of Pediatric Research at the European Medicines Agency (Enpr-EMA) aims to describe the research ethics review requirements including any pediatric specific requirements, as well as current or upcoming changes across six jurisdictions – the European Union (EU), United Kingdom (UK), United States of America (USA), Canada, Japan, and Australia.

**Methods:**

An open questionnaire developed by the WG and directed at both the Competent Authorities (CA) and the national pediatric clinical trial networks arranged by jurisdictions.

**Results:**

A synopsis of the current regulatory requirements covers centralized versus independent review, comparisons between investigator initiated and industry sponsored clinical trials, timelines, review board members requirements and the consenting/assent process for clinical trial (CT) applications, application submission processes and application requirements for each of the six jurisdictions. It also describes changes currently or soon to be implemented in some jurisdictions.

**Conclusion:**

This environmental scan highlights the differences in ethics review for CTs in pediatric medicine development across six jurisdictions. While there is a growing trend for centralized ethics review, it is not universally permitted due to institutional, state/provincial, or national policies. Even where central review is allowed, local review may still be required for vulnerable populations like children. Harmonized and centralized ethics reviews offer advantages such as expert pediatric reviewers and efficient and consistent evaluations.

## 1 Introduction

Clinical drug trials (CTs) in children are often conducted as multi-jurisdictional, multicentre trials. This is necessary due to the small patient populations, the rarity of the diseases and limited specialty facilities. These multi-site and multi-jurisdictional studies provide the opportunity to execute well-powered trials; however, they require significant expertise and resources. Additionally, CTs need a separate competent authority (CA or Regulatory Authority) and at least one (and often multiple) ethics committee (EC) reviews in each participating jurisdiction. Assessment of the CT by ECs is crucial to guarantee the safety and well-being of the participants, particularly of vulnerable populations like children. However, multi-site studies requiring at times applications to each local EC for the exact same protocol adds to the overall costs and delays the initiation of CTs. The same study undergoing review by multiple ECs has consistently been reported as an impediment to the efficiency and the consistency of the process (1, 2). In addition to increased global multi-stakeholder collaboration, sustainable trials infrastructure with pediatric expertise, integration of trials as part of clinical care, and alignment of the pediatric research agenda with health priorities, harmonization of regulatory reviews is recognized as critical to enhance the global capacity to conduct pediatric CTs (3).

To streamline the research ethics review process, several jurisdictions have recently modernized or are in the process of modernizing their approaches. An accompanying article in the same issue of this journal describes the Clinical Trial Application process and the respective jurisdictional regulatory requirements from a child health perspective (4). This second article focuses on the similarities and differences, as well as the jurisdiction-specific guidance for the research ethics review process for pediatrics CTs. It presents recent or anticipated changes in legislation / regulation and expected implementation timelines and recommendations from the Enpr-EMA Working Group on international collaborations. The article has three main objectives: to assist investigators and industry sponsors in conducting multi-jurisdictional CTs in children, to identify regulatory and ethical challenges in conducting these trials on an international scale and to foster and enhance international collaboration.

## 2 Materials and methods

In 2007, the Pediatric Cluster was established by the European Medicines Agency (EMA) in the EU and the US Food and Drug Administration (FDA), as a forum to discuss the approach to pediatric development, pertaining to specific products, product classes or therapeutic areas to enhance the science of pediatric CTs and inform the pediatric development plans submitted to both agencies. Over the following years, the Pharmaceuticals and Medical Devices Agency (PMDA), Japan, Health Canada (HC), and the Therapeutic Goods Administration (TGA), Australia, joined the Pediatric Cluster. The five CAs meet on at least a monthly basis to discuss all aspects of pediatric development, including trial design, ethics, safety, and pediatric study feasibility. In 2018, building on this long-standing international collaborative exchange, the European Network of Pediatric Research at the European Medicines Agency (Enpr-EMA) established a Working Group (WG) on international collaborations made of the same five CAs and the national pediatrics clinical trials networks of the corresponding jurisdictions with the specific aim to facilitate the unique needs of pediatric studies in a multi-country, multisite and multijurisdictional setting. In addition, after the United Kingdom (UK) left the EU on the 31st of January 2020, the UK regulator (Medicines and Healthcare Products Regulatory Agency, MHRA) and two UK pediatric research national networks joined the working group.

The following networks participated in the survey:

- EU: Enpr-EMA and Conect4Children (one of the Enpr-EMA member networks).
- USA: Pediatric Trials Network at Duke University and I-ACT (Institute for Advanced Clinical Trials in Children).
- Australia: Australian Network of Pediatric Trial Centres (ANPTC).
- Japan: Japanese Pediatric Society Drug Development Network (JPedNet).
- Canada: Maternal Infant Child and Youth Research Network (MICYRN).
- UK: (National Institute for Health Research Clinical Research Network-Children, NIHR CRN-Children, and the National Health Services Scottish Children’s Research Network, NHS Sco CRN).

This Enpr-EMA WG conducted an environmental global scan across jurisdictions using an open questionnaire directed at both the CA and the Networks arranged by jurisdictions under the following four headings (the first three are addressed in the accompanying article in the same issue of this journal).

- Clinical Trial Regulatory Requirements.
- Clinical Trial Application Submission Process.
- Clinical Trial Application Requirements.
- Clinical Trial Research Ethics Review Requirements.

The WG conducted an environmental scan across jurisdictions using an open questionnaire directed at both the CA and the networks organized by jurisdictions. In addition to topics like clinical trial regulatory requirements and the application submission process (results presented in accompanying article), the questionnaire included questions related to clinical trial site ethics requirements.

The topics surveyed were chosen to address the core elements of clinical trial authorization processes across different jurisdictions. Including these areas enabled the working group to analyze critical regulatory and procedural similarities and differences that could impact the implementation and harmonization of pediatric clinical trials internationally. Insights gained from the survey responses could help inform strategies for streamlining cross-border approvals, and assess the feasibility of adopting risk-based approaches to regulation. It took approximately 2 years to complete and obtain the survey (2020/21). The delay in survey completion was the result of the high-level clearance that was required from the CAs for some of the jurisdictions, compounded by the fact that the survey was being completed in the initial parts of the pandemic. After the UK left the EU, the same questionnaire was completed by the UK regulator (MHRA) and the two UK Pediatric research national networks. All responses were reviewed together by the respective jurisdiction’s CA and pediatric trial network(s) to ensure agreement in responses. In drafting this manuscript, participants were repolled (2024) to confirm any new updates or changes in status of planned updates to their jurisdiction’s practices.

For each of the jurisdictions, a summary of the status is provided, as well as changes currently being implemented or to be implemented in the near future.

## 3 Results

### 3.1 Clinical trials ethics requirements

A synopsis of the current status of the ethic review in each jurisdiction is provided in Tables 1, 2. The summary includes information regarding: centralized vs. independent review, comparisons between investigator initiated vs. industry sponsored clinical trials, timelines, requirements of review board members and the consenting/assent process.

**Table 1:** Overview of research ethics review processes and streamlining initiatives across multiple jurisdictions.

Country	Centralized ethics review process- investigator-initiated clinical trials	Centralized ethics review process- industry-sponsored clinical trials	Ethics harmonization process	Proposed changes to ethics review process in the future	Ethics review timelines for clinical trial approval (number of days)
Australia	National Mutual Acceptance (NMA) for 6 participating states/territories	NMA	National Mutual Acceptance (NMA) for 6 participating states/territories	Yes: one stop shop plans for National trial acceptance and coordination	
Canada	No	No	Provinces of Quebec, Ontario, Alberta, and British Columbia have intra-provincial ethics review processes with interinstitutional agreement for delegation of review	Canadian Institutes of Health Research (CIHR) has recently put forward a grant to fund a 5-year national ethics harmonization process for multi-province, multi-site studies with the goal of achieving a single ethics review for pediatric research studies	Average 10–12 weeks for provinces without centralized review process. Ontario centralized stream: less than 1 month Quebec: average 74 days
EU	Yes (at National/Member State level)	Yes (at National/Member State level)	Centralized review of Clinical Trial Application. Ethics review is coordinated by each Member State at national level. Ethics committees are mostly involved in the part II assessment that remain of competence of the individual Member States concerned for the trial	The Clinical Trial Regulation (CTR) No 536/2014 came into application on 31 January 2022, repealing CT Directive 2001/20/EC	CTR sets common timelines for evaluation across all Member States, which is min.45 days.
Japan	Yes, but there are still many institutions that only allow their own ethics review.	Yes, but there are still many institutions that only allow their own ethics review.	The Ministry of Health, Labour and Welfare encourages centralized review.	No	Ethics review timeline of the Pediatric Clinical Trial Network, Japan is within 30 days. It varies in other review boards.
United States	Yes (for certain federally conducted or supported research)	Yes (for certain federally conducted or supported research)	Centralized Review by a single IRB of certain federally conducted or supported multi-site research conducted in the US, with some exceptions.	FDA has published two proposed rules that, if finalized, would include changes to the agency’s regulations regarding obtaining and documenting informed consent from research participants, amend the regulations governing institutional review board (IRB) membership and functions, and require the use of a single IRB review process for FDA-regulated multi-site research conducted in the U.S., with some exceptions (ref below).	IRB approval times are variable and typically range from 30 to 120 days
UK	Yes	Yes	Centralized Review	Following public consultation on legislative proposals for clinical trials, published 21 March 2023, there is a commitment to introduce more streamlined and efficient application processes, making it easier to apply for trials in the UK but without compromising on safety standards, by legislating for a combined MHRA/research ethics review, with internationally competitive approval timelines and more flexibility for sponsors to respond to questions raised by regulators. Yes, the current Medicine for Human Use (CT Regs) 2004 as amended UK being revised for a more streamlined process	Maximum time from submission to outcome from Ethics and MHRA is 60 days, unless the user asks for more time.

**Table 2:** Research ethics review processes across multiple jurisdictions with pediatric-specific considerations.

Country	Country acceptance of for-profit commercial ethics review board	Review process for publicly vs. privately funded clinical trials	Inclusion of at least one member knowledgeable in pediatrics/child health	Assent requirements for included children	Dual signatures for consent/assent forms
Australia	No, NMA currently applies to publicly funded health services across jurisdictions. No for profit HRECs currently registered for pediatric studies	Same	Human Research Ethics Board must be accredited and registered for pediatric research	Not applicable “Assent” not recognized by the National Statement Most institutions require consent if the mature minor parallel with parental/carer consent	Only if determined by the review body. Most will adopt a family centric approach.
Canada	No	Same	No	No formal requirement, typical administration for ages 7–14 but can vary depending on the province	No
EU	No	Same	Yes	Varies depending on country	Varies depending on country
Japan	Not common. Some site management organization have for-profit ethics review boards.	Same. The review process for clinical trials intended for regulatory approval differs from the review process for other clinical trials.	Not a regulatory requirement.	Not required, but recommended. School age children and older (7 years and older) based on the Q&A for ICH E-11.	Generally no. Only if determined by the review body
United States	Yes. The review and approval of research by for-profit commercial IRBs is permitted	Non-exempt federally conducted or supported research subject to the Common Rule must go through an IRB registered with the federal government and follow the regulations at 45 CFR part 46 (or analogous regulations, depending on the federal department or agency conducting or supporting the research). FDA-regulated clinical investigations must go through a registered IRB and follow applicable regulations, including, 21 CFR parts, 50, 56, 312, and/or 812. If a study is both federally conducted or supported and involves an FDA-regulated product, it may be subject to both FDA regulations and the Common Rule.	FDA regulations and the Common Rule require that the IRB be sufficiently qualified, including through the expertise and experience of its members to promote respect for its advice and counsel in safeguarding participants. If an IRB regularly reviews research that involves a vulnerable category of subjects, such as children, consideration must be given to the inclusion of one or more individuals who are knowledgeable about and experienced in working with those subjects.	Need for assent determined by the IRB and based on the capability of the child	For studies with greater than minimal risk that offer no prospect of direct benefit to the individual child, the permission of both parents is generally required. IRBs may determine that the permission of one parent is sufficient for studies involving no greater than minimal risk or that offer the prospect of direct benefit.
UK	Yes	Same	Yes	From approximately 7 years to 15 years	

### 3.2 Changes currently ongoing or soon to be implemented in some jurisdictions

Several jurisdictions have recently modernized their laws, regulations and policies to streamline research ethics review. For instance, the EU has implemented the Clinical Trials Information System (CTIS), and Health Canada is exploring a proportional risk-based framework. In the UK, combined review processes expedite ethics and regulatory approvals. These changes aim to support innovative trial designs, enable rapid responses to public health needs, and encourage global trial harmonization, ultimately fostering faster access to pediatric treatments.

#### 3.2.1 European Union

The EU has taken measures to support the streamlined review of pharmaceutical clinical trials. Until recently, multi-EU-country CTs required submission of a clinical trial application to at least one certified research EC in each country, with committees accredited under national/regional legislation. A centralized process was adopted in 2014 and came into application on the 31st of January 2022, with the launch of the EU portal and database, named as the Clinical Trial Information System (CTIS) for the CTA submission, supervision, and authorization, as well as communication of clinical trial information, for both commercial and non-commercial sponsors (5).

The application process includes the submission of two parts (6); Part I of the clinical trial application dossier consisting of the product monograph, scientific rationale, protocol, and therapeutic and safety aspects which is assessed jointly by the EU/EEA Member States concerned, and coordinated by one reference member state (RMS). The European Economic Area (EEA) include the 27 EU countries and also Iceland, Liechtenstein and Norway (Switzerland is not an EU and EEA country but is part of the single market under the European Free Trade Association). Part II of the dossier includes nationally required documentation of ethical nature (patient information, informed consent, suitability of investigator and investigational site), and is assessed individually by each Member State concerned, for each trial. The conclusions for both part I and part II are required within a defined timeline and constitute the basis for the Member States decision (individual decision) on the authorisation of the trial application. The Clinical Trial Regulation does not specify which part of the application is reviewed and assessed by the national CA or EC, it is up to each EU/EEA country to coordinate the assessment at the national level (5). The CTIS is maintained by the EMA.

CTs involving pediatric participants in the EU are subject to specific ethical requirements and regulatory considerations distinct from those applied to adult trials. These requirements are designed to protect the heightened vulnerability and unique needs of children and adolescents in clinical research. For pediatric trials, a more stringent consent process is mandated. Informed consent must be obtained from the legal guardian(s) of the child, but the child must also be involved in the decision process in an age-appropriate way. Assent (agreement to participate) should be sought from children who are able to understand the trial in a developmentally appropriate manner. This process respects the autonomy of children by allowing them to be part of the decision. The risk–benefit analysis for pediatric participants is stricter than for adults. Clinical trials involving children can only proceed if they present a direct benefit to the children participating, and minimal risk levels are generally expected unless justified by potential direct benefits to the child. Finally, pediatric trials require review by ethics committees that include expertise in pediatric medicine or child-specific ethics. This requirement ensures that the specific needs, risks, and ethical concerns for children are adequately considered.

#### 3.2.2 Canada

Research Ethics Boards (REB) in Canada have historically been located within each institution rather than centrally. To help reduce disparities among REB outcomes and standardize REB review processes, there have been several proposals to initiate or explore a national program of accreditation (7–10). Although several provinces (Ontario, Quebec, Alberta) have made significant progress in streamlining research ethics review within their jurisdictions, very little progress toward a single research ethics review across Canada occurred until a recent effort initiated by the Canadian Institute for Health Research (CIHR), the federal health research funder, resulting in the development of the Canadian Collaboration for Child Health: Efficiency and Excellence in the Ethics Review of Research (CHEER) (11). By the end of this national grant in 2026, the CHEER collaboration is expected to develop a cross-province streamlined ethics review process for multi-site studies aiming to achieve a single ethics review for pediatric clinical trials in Canada. The CHEER project has several deliverables: (1) Canada-wide streamlined research ethics review platforms customized to support the needs of the child health research community, considering different states of readiness and legislative and structural particularities; (2) an REB assessment program to ensure quality and consistency in research ethics review and engender trust across the REB and institutional communities; and (3) educational resources to advance the ethical conduct of child health research and support efficiency, quality and consistency in research ethics reviews. Streamlining ethics review in Canada is also one of the objectives of the Accelerating Clinical Trials (ACT) Canada Consortium. Although non exclusively focused on pediatric trials, the mandate of ACT, a 3-year (2022–2025) $39 million CIHR investment, is to accelerate, optimize, and facilitate the conduct, implementation and results translation from high-quality high-impact randomized controlled trials (12). The ACT consortium is funding CanReview, a multi-stakeholder group tasked to set up and run a pan-Canadian, distributive, single REB review and approval process with strict timelines for the initiation and conduct of multicentre CTs (12).

#### 3.2.3 United Kingdom

The MHRA and the Department of Health in Northern Ireland, working closely with the Health Research Authority (HRA), consulted on a set of proposals to update, improve and strengthen the UK legislation that underpins the regulation of clinical trials. The outcome of the consultation and the government’s proposals were published in March 2023 (13). A key proposal was to introduce a combined regulatory and ethics approval process into legislation, while keeping the option for independent submissions available for rare exceptions. This approach aims to create a proportionate and flexible regulatory environment that is streamlined, agile and responsive to innovation, while assuring participant safety. The MHRA will continue to make enhancements with new legislative measures to make it easier and faster for applicants to gain approvals and to ensure the UK remains a prime destination for clinical trials (14). More detailed information on the planned overhaul of UK clinical trials regulations were recently (December 2024) made available (15).

#### 3.2.4 United States of America

In recent years, the U.S. government has introduced several important changes aimed at modernizing, strengthening, and streamlining the research ethics review process. These changes impact multi-site research involving children and reflect the growing complexity and scale of clinical investigations. Through updated regulations, policies, and guidance, federal departments and agencies have sought to improve efficiency, promote ethical standards, and ensure that human subjects in research, including children, are adequately protected. Key updates from the U.S. Department of Human Health and Services (HHS), the National Institutes of Health (NIH), and FDA, illustrate the evolving landscape of research oversight in the U.S.

In January 2017, the HHS and 15 other U.S. federal departments and agencies issued a final rule, that was further amended on the 22nd of January and 19th of June 2018, to update the Federal Policy for the Protection of Human Subjects, also known as the Common Rule (16–18). The revisions to the Common Rule included a new requirement that any institution located in the U.S. that is engaged in cooperative research (i.e., multi-site research) conducted or supported by a Federal department or agency subject to the Common Rule rely upon approval by a single Institutional Review Board (IRB) for that portion of the research that is conducted in the U.S., with some exceptions (19). The compliance date for the single IRB review requirement for cooperative research (for both pediatric and/or adult populations) was the 20th of January 2020.

On the 25th of January 2018, the U.S. National Institutes of Health (NIH) implemented a policy establishing the expectation that all domestic sites of multi-site studies funded by the NIH and conducting the same protocol involving non-exempt human subjects research be reviewed by a single IRB, with some exceptions (20). The NIH is not involved in the selection of the IRB, which may be a single IRB (usually already existing) selected on a study-by-study basis, a central IRB established for a specific purpose/projects, or an independent “commercial” (or private) IRB. The plan for single IRB review is expected to be included in the application for funding and agreed to by all participants through reliance agreements. An NIH-funded multi-site study being conducted at more than one domestic site may be subject to the NIH Single IRB policy and/or the Common Rule’s cooperative research provision (21).

To ease common challenges associated with initiating the single IRB review process in the U.S., the NIH National Centre for Advancing Translational Sciences funded a project to establish a national IRB reliance network that would support national adoption of a single IRB review. The Streamline, Multisite, Accelerated Resources for Trials (SMART) IRB platform was launched in 2016. More than 1,200 institutions have voluntarily joined SMART IRB’s Master Common Reciprocal IRB Authorization Agreement and use the SMART platform to support inter IRB arrangements. The platform also provides supportive tools and resources to aid institutions in complying with single IRB review (22).

On the 28th of September 2022, FDA published two proposed rules that, if finalized, would harmonize certain sections of FDA’s regulations on human subject protection and IRBs, to the extent practicable and consistent with other statutory provisions, with the revised Common Rule. One of the proposed rules (23) includes potential changes to FDA’s regulations regarding obtaining and documenting informed consent from research participants, and IRB membership and functions, including continuing review. The other proposed rule (24) concerns a potential change to FDA’s regulations that would require any institution located in the U.S. participating in FDA-regulated multi-site research to rely on approval by a single IRB for that portion of the research that is conducted in the U.S., with some exceptions. Although the proposed rules would harmonize FDA’s human subject protection requirements, to the extent practicable and consistent with statutory provisions, with those of the revised Common Rule, some aspects of the proposed rules are necessarily different from the revised Common Rule due to the different scope of research that FDA regulates. For example, FDA has proposed several exceptions from the single IRB review requirement that are different from the exceptions to the revised Common Rule’s single IRB review requirement (24). In the preamble to the proposed rule, FDA explained that the different proposed exceptions reflect circumstances for which FDA believes requiring the use of a single IRB for oversight of multi-site research may not be appropriate for FDA-regulated research. Recognizing the unique ethical challenges of pediatric research, FDA release a draft guidance in September 2022, entitled “Ethical Considerations for Clinical Investigations of Medical Products Involving Children.” The draft guidance describes the ethical framework in FDA’s regulations for clinical investigations involving children and describes the application of Title 21 of the Code of Federal Regulations (CFR) part 50, subpart D to pediatric clinical investigations regulated by FDA. When finalized, the draft guidance will represent FDA’s current thinking regarding ethical considerations for clinical investigations of drugs, biological products, and medical devices involving children (25).

Efforts to ensure that informed consent documents and consent process in FDA-regulated studies satisfy the FDA regulatory requirements were further reinforced in August 2023, with the issuance of a new guidance for IRBs, clinical investigators, and sponsors. The guidance provides the Agency’s current recommendations regarding informed consent and describes FDA regulatory requirements for informed consent and addresses frequent questions, including those related to enrolling children in clinical investigations (26, 27). Collectively, the actions taken by the FDA, and future planned actions, are intended to contribute to making informed consent documents and the consent process more informative, fit for purposes, and participant centered/partnered.

As part of its efforts to implement provisions of the 21st Century Cures Act, FDA issued a final rule in December 2023 that amends its regulations to allow an exception from the requirement to obtain informed consent when a clinical investigation poses no more than minimal risk to the human subject and includes appropriate safeguards to protect the rights, safety, and welfare of human subjects. The final rule permits an IRB to waive or alter certain informed consent elements or to waive the requirement to obtain informed consent, under limited conditions, for certain FDA-regulated minimal risk clinical investigations including those involving children (27).

#### 3.2.5 Japan

Clinical trials for drug approvals (“Chiken” in Japanese) must be performed under the Japanese Pharmaceutical and Medical Devices Act. There is a clear distinction between “Chiken” and other types of research (28). Previously separate guidelines and laws were integrated into the new Ethical Guidelines for Medical and Biological Research Involving Human Subjects in June 2021, and three major changes were introduced: centralized review of clinical trial applications, electronic informed consent, and research cooperating organization (29). Centralized review is not mandated in these guidelines. On the other hand, there are two laws that mandate centralized review for certain types of clinical trials. The Act on the Safety of Regenerative Medicine mandates centralized review for clinical trials or treatment using unapproved or off-label regenerative medicines (30). The Clinical Trials Act mandates centralized review for specified clinical trials that either use (1) unapproved or off-label medicines or (2) the investigators receive research funds or other benefits from the manufacturer that holds marketing approval of the study drug (31).

Central review boards mandated by the Act on the Safety of Regenerative Medicine are different from the ones mandated by the Clinical Trials Act, and they are different from the ethics review boards for “Chiken” or other clinical trials. Furthermore, ethics review boards for “Chiken” are different from the ones for other clinical trials governed by the Ethical Guidelines for Medical and Biological Research Involving Human Subjects. This is probably the reason why Japan has so many ethics review boards compared to other countries (32).

There is currently ongoing discussion regarding a revision of the Clinical Trials Act. One of the major discussion points related to pediatrics is the definition of “off-label” medicines in relation to the designation of specified clinical trials. In the recent discussion in Japan, “off-label” use in a strict sense is considered to be the use outside the approved indication and/or in contraindicated conditions. However, some ethics review boards consider the study drugs “off-label” in a broader sense when the safety of the drug has not been established in certain age groups and/or there is no clear pediatric dosage on the label, even when the drugs are commonly prescribed in children. In that case the clinical trials with these drugs must be conducted under the Clinical Trials Act. Some measures are expected to be introduced to eliminate variation in judgments on “off-label” use in the forthcoming revision of the Act.

#### 3.2.6 Australia

Clinical trials involving “unapproved” therapeutic goods must be conducted in accordance with the requirements of the therapeutic goods legislation (the Therapeutic Goods Act 1989), the principles that have their origin in the World Medical Association Declaration of Helsinki, the Australian National Statement on Ethical Conduct in Human Research (the National Statement) as in force from time to time, the relevant Good Clinical Practice (GCP) guideline, other relevant requirements of Commonwealth and/or state and territory legislation as well as site specific requirements (33). Human Research Ethics Committees (HRECs) are required to be registered, and to comply with the requirements as set out in the National Statement, with additional criteria for pediatric HRECs (33). Future development of the national one-stop shop will enable streamlined clinical trial cross-jurisdictional conduct. The aim is to better enable patients, researchers, industry representatives and sponsors to find, conduct, participate and invest in high quality and ethical research in Australia (34).

## 4 Discussion

Identifying areas where there might be agreement in policies and where standards might be defined has the potential to improve the ethics review process for CTs involving children, thereby expediting access to these trials. The survey highlighted the differences between ethics review for CTs in pediatric medicine development among six jurisdictions (Australia, Canada, EU, Japan, UK, US).

Most jurisdictions, including the EU (at national level of each Member State), USA, UK, Japan, and Australia (via the National Mutual Acceptance (NMA) system), have some form of centralized ethics review, though Canada’s system is still largely provincial. Canada is working toward a national harmonized process for multi-site studies, while the USA and UK are advancing streamlined review procedures. Although there is a growing trend for ethics review to be performed centrally, this is not universally permitted. Furthermore, within some jurisdictions, the use of centralized ethics review may not be allowed because of institutional or national policies. In cases where central review is permitted, clinical sites may require local review for vulnerable populations (e.g., children). The WG highly recommends the use of central ethics reviews, noting that centralized ethics reviews have some clear advantages such as the availability of expert pediatric reviewers and the efficiency and consistency of the review, for example.

Informed consent provided by a parent, guardian and/or legally authorized representative is universally required in all jurisdictions (35). Regarding assent, most jurisdictions acknowledge the importance of obtaining it from children who can understand the trial. The practice of assent, whereby children developmentally capable of understanding the trial must receive age-appropriate information from trained professionals, and their explicit wishes to refuse or withdraw from participation must be respected is unique to pediatric populations and is derived from the important ethical principle of autonomy. In Europe, the Part II assessment in the CTIS is dependent on each Concerned Member State, having its own national legislation and detailed requirements for the ethical assessment documents. This variation can create some extra work or delays for sponsors, if there is a need for document adaptation or amendments for several countries. To tackle these ethical issues and to support sponsors, Enpr-EMA have developed specific guidance for pediatric trials (36). The importance of recognizing patient voices and rights is increasingly emphasized in clinical trials, with special attention to including the perspectives of pediatric participants (37). It is notable that assent is typically legally required (EU, UK, USA and Canada) or strongly encouraged (Australia, Japan) across all jurisdictions. The general recommendation is to obtain assent from children aged 7 years and older. Given the importance of respecting the rights of children, the WG strongly recommends obtaining assent from pediatric trial participants who are developmentally capable to do so.

In summary, while the ethics review processes and requirements for pediatric trials vary globally, the WG emphasizes the importance of centralized review systems, pediatric expertise, and child assent in ways that aim to protect young trial participants while respecting their emerging autonomy. Each country’s approach reflects a balance between ensuring safety and supporting efficient research practices, with ongoing efforts in several jurisdictions to streamline and harmonize ethics review for pediatric clinical trials.

## Data Availability

The original contributions presented in the study are included in the article/supplementary material, further inquiries can be directed to the corresponding author.
