# Erratum: Requirements and special considerations for drug trials with children across six jurisdictions: 2. Ethics review in the regulatory approval process

**DOI:** 10.3389/fmed.2025.1611335

**Published:** 2025-04-29

**Authors:** Frontiers Production Office

**Affiliations:** Frontiers Media SA, Lausanne, Switzerland

**Keywords:** pediatrics, clinical trials, regulatory science, research ethics review, research ethics committee, institutional review board

Due to a production error, there was an error in the Conflict of Interest Statement. Instead of “The authors declare that the research was conducted in the absence of any commercial or financial relationships that could be construed as a potential conflict of interest.” It should have read “DS is currently employed by company WCG Clinical.

The remaining authors declare that the research was conducted in the absence of any commercial or financial relationships that could be construed as a potential conflict of interest.”

The publisher apologizes for this mistake.

The original article has been updated.

